# The use of SCL-k-9 to measure general psychopathology in women and men with skin conditions

**DOI:** 10.3389/fpsyg.2022.977264

**Published:** 2022-10-20

**Authors:** Tonia Samela, Giorgia Cordella, Valeria Antinone, Paride Sarandrea, Anna Rita Giampetruzzi, Damiano Abeni

**Affiliations:** ^1^Clinical Epidemiology Unit, IDI-IRCCS, Rome, Italy; ^2^Clinical Psychology Unit, IDI-IRCCS, Rome, Italy; ^3^Dermatology Unit, IDI-IRCCS, Rome, Italy

**Keywords:** skin diseases, patient-reported measures, psychological screening tool, gender differences, psychology in clinical settings

## Abstract

**Objectives:**

To measure general psychopathology in dermatologic outpatients using the Symptom-Checklist-K-9 (SCL-K-9); to investigate whether the SCL-K-9 is able to categorize patients with and without significant non-psychotic disorders; and to perform a single-item analysis of the SCL-K-9, with a focus on gender differences.

**Methods:**

Cross-sectional study on consecutive dermatological patients. We used two self-administered questionnaires to assess general psychopathology symptoms: General Health Questionnaire-12 (GHQ-12) and SCL-K-9. Sociodemographic information was collected with standardized forms. The performance of the SCL-K-9 in classifying patients according to their current emotional distress severity was assessed using a ROC procedure. Finally, we measured differences in scores obtained among women and men in SCL-K-9 single items.

**Results:**

A total of 292 patients were studied (71.2% women). We observed statistically significant differences in SCL-K-9 total mean scores and in most single items among genders. We found that it would be more appropriate to use gender-specific cut-offs when using SCL-K-9 to screen dermatological patients for general psychopathology.

**Conclusion:**

The SCL-K-9, with its compact format could provide, in a short time, a wide range of information related to critical areas that challenge the mental health of patients with skin diseases.

## Introduction

Different dermatologic conditions are chronic, relapsing, difficult to manage and characterized by a wide variety of symptoms. Although skin conditions, even the severe ones, are not often directly life-threatening, it has been reported that dermatology patients are at greater risk of experiencing mental illness than the general population (Picardi et al., 2000; Huilaja et al., 2018; Konstantinou and Konstantinou, 2019), and the co-existence of psychological distress and chronic skin conditions is well established in the literature (Sampogna et al., 2004; Picardi et al., 2006a; Konstantinou and Konstantinou, 2019; Jafferany et al., 2020; Mento et al., 2020; Finlay et al., 2021).

Several studies are also stressing the observation that women sustain a greater burden of illness compared to men with skin diseases (Ständer et al., 2013; Napolitano et al., 2020), or autoimmune diseases with skin manifestations (Ngo et al., 2014) or other chronic conditions (August and Sorkin, 2010; Kim et al., 2011; Kim and Kim, 2018).

For these reasons a general personality and psychopathology evaluation should be considered a crucial part of the multidisciplinary assessment for dermatologic issues (Panebianco et al., 2018), and it would also be advisable to pay greater attention to gender differences in the process of evaluation.

However, as confirmed by a multi-center study conducted across 13 countries in Europe, dermatologists would tend to underestimate psychiatric symptoms in a significant group of dermatologic patients (Dalgard et al., 2018), for reasons that could be related to the limited availability of time and to the high number of patients attending the clinical units daily.

Several tools have been validated in order to measure psychological distress in dermatologic outpatients, with consistent results (Lewis and Finlay, 2004; Picardi et al., 2004; Prinsen et al., 2010; Sampogna et al., 2013); therefore, quick and manageable self-reported screening tools are available, and could be useful for clinicians in order to identify patients who need a more in-depth psychological assessment procedure, and possibly counselling and support.

The Symptom Checklist-90-Revised (SCL-90-R; Derogatis and Savitz, 2000) is a commonly used self-rating measure, composed by ninety items, for assessing general psychopathology in different populations (Schauenburg and Strack, 1999; Hardt et al., 2000; Bianciardi et al., 2021; Castelo-Branco et al., 2021; Melchior et al., 2022; Timman and Arrindell, 2022). Additionally, this tool has demonstrated a factorial invariance (i.e., the concept that postulates that the psychometric properties of a questionnaire, used either by multiple groups or by the same group over time, have to be identical to ensure an unbiased comparison of factor means) across genders (Derogatis and Cleary, 1977).

Since the 1980s, several short forms of the SCL-90-R have been proposed to provide a more manageable tool (Prinz et al., 2013). Among these versions, the Symptom-Checklist-K-9 (SCL-K-9), composed of the nine items of the SCL-90-R (i.e., #24, #28, #31, #34, #43, #57, #58, #75, #77) that exhibit the highest item-total correlation, was proposed as an efficient screening tool representing all of the original symptom subscales of the SCL-90-R (Klaghofer and Brähler, 2001). It was also validated in the German (Klaghofer and Brähler, 2001) and Ukrainian (Sereda and Dembitskyi, 2016) general populations, and in Italian overweight and obese patients seeking weight-loss treatment (Imperatori et al., 2020). Furthermore, Petrowski et al. (2019), also confirmed results of factorial invariance of the scale across gender groups in representative German population (Petrowski et al., 2019).

Therefore, the SCL-K-9 could be considered a suitable and valid tool that could be used within the time restraints that preclude the use of the full-length form in the busy daily clinical practice.

To the best of our knowledge, no study has ever applied this tool to measure general psychopathology in dermatologic patients during the routine clinical activities. Thus, the aims of the present study were: (i) to measure general psychopathology in dermatologic outpatients using SCL-K-9; (ii) to investigate, using a receiver operating characteristic (ROC) curve procedure, whether the SCL-K-9 is able to categorize patients with and without significant non-psychotic disorders (i.e., serious emotional distress; Piccinelli et al., 1993) using the GHQ-12 as the standard test; and (iii) to perform a single-item analysis of the SCL-K-9, with a focus on gender differences.

## Materials and methods

### Study design, setting, and participants

This is a cross-sectional, single-center observational study. The research has been conducted in accordance with the Declaration of Helsinki and was approved by the Institutional Ethical Committee. Data were collected from October 7, 2021 to May 9, 2022 at the dermatological research hospital IDI-IRCCS, Rome, Italy. Specifically, consecutive patients were enrolled in the Dermatological Day-Hospital Unit, the inpatient Dermatological Clinic, and the Outpatient Dermatological Unit of IDI-IRCCS. Study patients had to satisfy the following inclusion criteria: (i) ≥18 years old; (ii) a clinical diagnosis of a chronic or recurrent skin condition; and (iv) signed written informed consent. Exclusion criteria were: (i) inability to understand Italian; (ii) inability to understand the questionnaires; and (iii) any diagnosed major neuro-psychiatric disorder (i.e., diagnosed major depressive disorder, bipolar disorders, schizophrenia, attention deficit disorder, eating disorders, addictions, mild–severe cognitive impairment, and epilepsy or seizures).

### Disease severity outcome measure

The Physician Global Assessment (PGA) is a 5-point scoring system used to assess disease severity (Pascoe et al., 2015).

### Patients’ self-report measures

After enrolment, the Italian version of the SCL-K-9, and the 12-item General Health Questionnaire (GHQ-12) were distributed to all patients. Both tools are self-report.

The SCL-K-9, as already described above, is the 9-item screener for global psychological symptom severity (Imperatori et al., 2020). Items are scored on 5-point Likert scale, with range “0” to “4,” investigating the severity of nine main psychopathological dimensions over the week before the survey, including: somatic symptoms, interpersonal sensitivity, obsessive–compulsive behaviors, anxiety and depressive symptoms, hostility, phobic symptoms, paranoid tendencies, and psychoticism. This scale yields a global severity index (GSI) as a measure of general psychopathology severity, with higher scores reflecting higher levels of psychopathological distress as well as a greater severity of self-reported symptoms.

The GHQ-12 is a self-report questionnaire that measures psychological distress and may detect current non-psychotic psychiatric disorders (Piccinelli et al., 1993). GHQ-12 scores were computed both through the dichotomous scoring method (0–0–1–1), and Likert scoring method (Picardi et al., 2000) in order to detect “GHQ-cases” (i.e., a GHQ score ≥ 7 is indicative of the presence of probable and clinically relevant depressive syndrome; Picardi et al., 2004, 2005a,b). It is a validated tool widely used in dermatological research (Abeni et al., 2002; Picardi et al., 2004). In this study the GHQ-12 “case” status has been used as the standard measure to evaluate SCL-K-9 performance.

Pain perception was assessed through the Visual Analog Scale for pain (VAS). VAS is a psychometric instrument designed to assess the level of pain related to symptom severity in patients, and it is used to achieve a statistically measurable and reproducible classification of symptom severity and disease control (Price et al., 1983). We used the 10 cm scale, with 0–10 as possible range.

### Data collection procedures

Patients were informed about the aim of the study and, after signing the written informed consent form, they completed the sociodemographic (i.e., gender, age, height and weight in order to assess body mass index, dermatologic diagnosis, duration of the disease, educational level expressed in school-years, marital status, employment) and the psychological self-report measures in a standardized form.

### Statistical analysis

The study sample corresponds to the actual number of patients seen during the study period who agreed to participate to the study; being a screening-based study, no sample size calculation was performed.

Categorical variables were described as number and percentage, and continuous variable were firstly classified in different levels and then described as discrete (Table 1).

**Table 1:** Sociodemographic and clinical features of the study participants, with SCL-K-9 total mean scores.

Variable	Level	*N* ^*^	%	SCL-K-9 tot.		
				*M*	*SD*	*p*
Overall		292	-	0.79	0.73	
Gender	Male	84	28.8	0.88	0.73	<0.001
	Female	208	71.2	0.56	0.73	
Age (median)	<53	143	49.8	0.85	0.77	0.143
	53+	144	50.2	0.72	0.70	
Education (years)	≤8	67	23.8	0.81	0.77	0.853
	9–13	155	55.0	0.78	0.73	
	14+	60	21.2	0.74	0.70	
Marital status	Single	81	28.6	0.93	0.85	0.088
	Married/widower	147	51.9	0.75	0.65	
	Other	55	19.5	0.71	0.69	
BMI (kg/m^2^)	<25	168	60.9	0.81	0.72	0.535
	25–29	73	26.5	0.70	0.70	
	30+	35	13.6	0.73	0.76	
Smoking	No	153	53.9	0.78	0.74	0.017
	Yes	78	27.5	0.93	0.80	
	Ex	53	18.6	0.56	0.54	
GHQ-12	<7	258	89.6	0.64	0.59	<0.001
	7+	30	10.4	2.03	0.71	
VAS	<7	140	55.8	0.60	0.58	<0.001
	7+	111	44.2	1.10	0.81	
Diagnosis	Dermatologic	135	46.2	0.80	0.77	0.025
	Autoimmune	114	39.0	0.87	0.72	
	In assessment	43	14.8	0.52	0.59	

* Totals may vary due to missing data.

*m*, means; *SD*, standard deviation; SCL-K-9, Symptom Checklist-K-9; BMI, Body Mass Index; GHQ-12, 12-item General Health Questionnaire; VAS, Visual Analogue Scale for Pain; Autoimmune, dermatologic autoimmune disease; Dermatologic, chronic, non-autoimmune skin condition; In assessment, under dermatologic diagnostic assessment.

**Figure 1:**
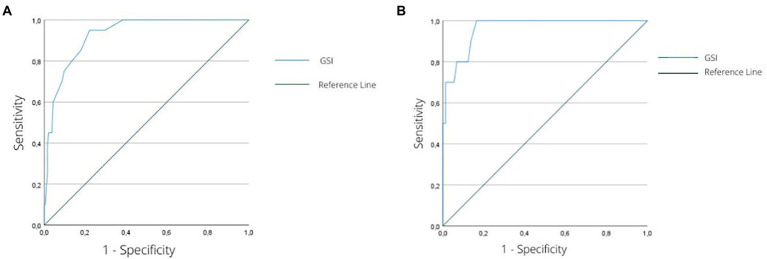
**(A)** ROC curve graph for the ability of the GSI to discriminate female individuals with significant psychological distress levels (GHQ-12 score 7+) from those without a significant level of psychological distress (GHQ-12 < 7). **(B)** ROC curve graph for the ability of the GSI to discriminate male individuals with significant psychological distress levels (GHQ-12 score 7+) from those without a significant level of psychological distress (GHQ-12 < 7). ROC, receiver operating characteristic; GSI, global severity index of the Symptom Checklist-K-9; GHQ-12, 12-item General Health Questionnaire.

Reliability was assessed using Cronbach alpha for all scales. To measure differences in scores obtained among women and men in SCL-K-9 single items and GSI, the mean and standard deviation scores were computed for each item, separately for males and females (Figure 1). Spearman’s correlation coefficient was used to measure correlation between the instruments’ total scores.

To evaluate the performance of the SCL-K-9 in classifying patients according to their current emotional distress severity, we performed a ROC test procedure (Ruopp et al., 2008). A ROC curve is a bidimensional description of test performances (Fawcett, 2006), with the main outcome variable being the area under the ROC curve (i.e., AUC), which reflects the probability that a randomly sampled respondent would be correctly categorized (Centor and Schwartz, 1985). The AUC value represents the overall accuracy of the instrument in indexing a sample (Swets, 1988), where values of ≥0.70 are classified as satisfactory. Additionally, the Youden Index (Youden, 1950) was calculated in order to detect the maximum thresholds of both sensitivity (i.e., the proportion of individuals who have the target condition and receive positive test results) and specificity (i.e., the proportion of individuals without the target condition who receive negative test results). The same procedure was also conducted by distinguishing the scores among women and men for the purpose of verifying the presence of different gender-specific cut-offs (Table 2).

**Table 2:** Proportion of participants, separately for women and men, who reach the SCL-K-9 proposed cut-offs.

Variable	Level	SCL-K-9 tot. ≥ cut-off					
Female			Male		
		*N* ^*^	% ≥1.277	*p*	*N* ^*^	% ≥0.720	*p*
Overall	-	205	28.8	-	84	26.2	-
Age (median)	<53	102	30.4	0.612	41	36.6	0.046
	53+	103	27.2		41	17.1	
Education (years)	≤8	45	28.9	0.997	22	27.3	0.378
	9–13	109	29.4		46	21.7	
	14+	45	28.9		15	40.0	
Marital status	Single	56	32.1	0.825	25	44.0	0.025
	Married/widower	45	26.7		10	20.0	
	Other	101	28.7		46	15.2	
BMI kg/m^2^	<25	134	26.1	0.371	34	26.5	0.621
	25–29	37	37.8		36	25.0	
	30+	26	30.8		9	11.1	
Smoking	No	118	26.3	0.268	35	25.7	0.671
	Yes	53	37.7		25	32.0	
	Ex	32	25.0		21	19.0	
GHQ-12	<7	185	22.2	<0.001	73	16.4	<0.001
	7+	20	95.0		10	100	
VAS	<7	92	19.6	0.002	48	20.8	0.023
	7+	85	41.2		26	46.2	
Diagnosis	Dermatologic	155	30.3	0.471	46	28.3	0.930
	Autoimmune	18	16.7		15	26.7	
	In assessment	33	27.3		21	23.8	

* Totals may vary due to missing data.

*m*, means; *SD*, standard deviation; SCL-K-9, Symptom Checklist-K-9; BMI, Body Mass Index; GHQ-12, 12-item General Health Questionnaire; VAS, Visual Analogue Scale for Pain; Autoimmune, dermatologic autoimmune disease; Dermatologic, chronic, non-autoimmune skin condition, In assessment, under dermatologic diagnostic assessment.

Finally, a SCL-K-9 single-item analysis was performed to portray the presence of psychopathological symptoms detected among women and men. The mean values of these two independent groups were then compared, using the non-parametrical Mann–Whitney *U*-test, to determine whether there was statistical evidence that the population means are significantly different (Table 2).

The results were considered significant if *p* < 0.05. Spearman r coefficients were reported as measures of association. Spearman r coefficients <0.10 are considered to be a negligible or null effect, r between 0.10 and 0.30 a small effect, r between 0.30 and 0.50 a medium effect, and r ≥ 0.50 a large effect (Cohen, 1988). All statistical analyses were run under IBM® Statistical Package for Social Science (SPSS), version 28.0 for Windows.

## Results

Two hundred and ninety-two patients took part in the study. There were 208 women (71.2%) and 84 men (28.7%); the median age was 53 years, and 40.1% had overweight or obesity (i.e., BMI ≥ 25); 27.5% were currently smoking. At the time of enrollment 46.2% of the sample had a non-autoimmune skin condition, 39.0% had a dermatologic autoimmune disease, and 14.8% definitely had a skin condition, but were still undergoing further diagnostic assessment. Concerning clinical features, 11.3% had a GHQ-12 score ≥ 7; 44.2% had a VAS score ≥ 7. The mean GSI score was 0.79 (SD ± 0.73). Other socio-demographic features of the sample are summarized in Table 1, which also shows the mean GSI scores in the different levels of the main variables of interest.

We observed a large and statistically significant difference in GSI mean scores among genders (0.88, for women vs. 0.56 for men; *p* < 0.001). Significant differences in GSI scores were also found in scores below and above the GHQ-12 cut-off (0.64 mean score for GHQ-12 < 7 vs. 2.03 mean score for GHQ-12 7+; *p* < 0.001). Moreover, the GSI scores were significantly different in pain perception (i.e., VAS), with 0.60 mean score for VAS <7 points and 1.10 mean score for VAS 7+ points (*p* < 0.001). The comparison in GSI score according to diagnosis was also significantly different: patients with a dermatologic-autoimmune disease reported a higher GSI score compared to patients with dermatologic disease, and to patients undergoing diagnostic assessment (i.e., 0.87, 0.80, 0.52 GSI mean score respectively; *p* = 0.025).

In the present sample Cronbach’s α were 0.87 for SCL-K-9 nine items, and 0.88 for GHQ-12 twelve items; therefore, the study measures can be considered reliable. Correlations between these two measures (i.e., GSI and GHQ-12 total Likert 0–36 score) were significantly and strongly associated (*r* = 0.68, *p* < 0.001), but did not overlap. Thus, it is possible to hypothesize that GHQ-12 and SCL-K-9 do not measure the same construct, detecting different forms of psychological distress in dermatological setting.

**Figure 2:**
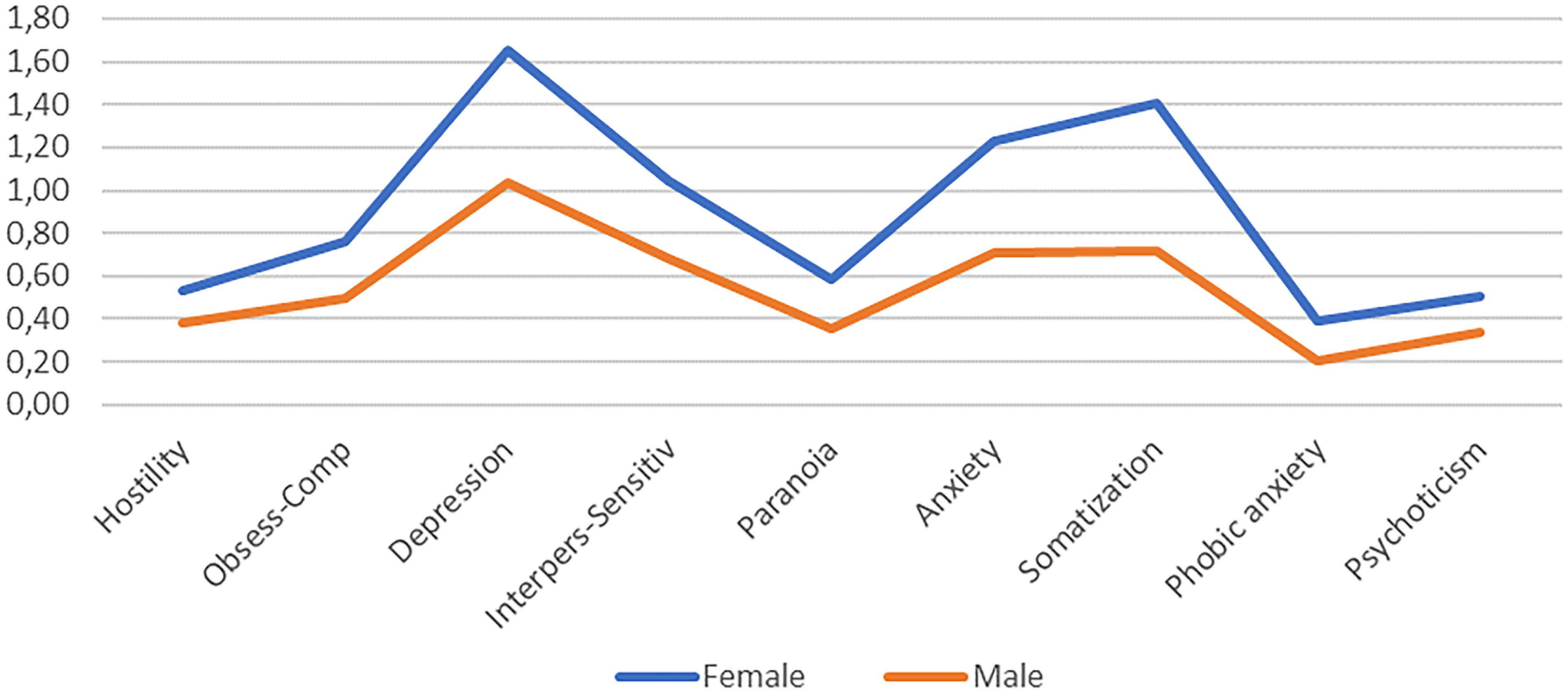
SCL-K-9 single-item analysis. Gender-specific GSI mean scores.

The ROC curve analysis showed that both the GSI score for women (area under the ROC curve = 0.92, 95%; confidence interval [0.88, 0.97], *p* < 0.001; Figure 1) and the GSI score for men (area under the ROC curve = 0.96, 95%; confidence interval [0.92, 1.00], *p* < 0.001; Figure 2) accurately classified patients with relevant psychological distress.

Particularly, for women, a score of 1.277 or higher on the GSI (Youden index = 0.73) categorized individuals with a sensitivity of 0.95 (95% of all the individuals with GHQ-12 > 7 were correctly detected) and a specificity of 0.78 (22% of patients were incorrectly identified as having a relevant psychological distress). The same procedure conducted for men revealed that a score of 0.720 of higher on the GSI score (Youden index = 0.84) classified patients with relevant psychological distress (i.e., GHQ-12 ≥ 7) with a sensitivity of 1.00 (100% of all the individuals with GHQ-12 ≥ 7 were correctly detected) and a specificity of 0.84 (16% of patients were incorrectly identified as having relevant psychological distress).

According to the new proposed cut-offs, 28.8% of women and 26.2% of men were above the GSI cut-offs. Among women, the significant differences were observed for GHQ-12 and VAS (i.e., 95.0% vs. 22.2%; 41.2% vs. 19.6%, respectively). Among men, in addition to GHQ-12 and VAS (i.e., 100.0% vs. 16.4%; 46.2% vs. 20.8%, respectively), younger (36.6% vs. 17.1%) and single patients (44.0%) had significantly higher proportions of above cut-off GSI score. The full description of sociodemographic features of the sample according to SCL-K-9 proposed cut-offs reported in Table 2.

The single-item analysis, using the non-parametrical Mann–Whitney U-test, showed that all the SCL-K-9 symptoms mean values were higher in women than in men. Specifically, women reported significantly higher scores indicative of psychological distress, in the symptomatic areas of hostility (*p* = 0.032), obsessive–compulsive symptoms (*p* = 0.004), depressive symptoms (*p* < 0.001), interpersonal sensitivity (*p* = 0.003), anxiety symptoms (*p* < 0.001), somatization symptoms (*p* < 0.001), and psychoticism (*p* = 0.020). Higher scores, among women, even if not statistically significant, were also observed in paranoid ideation symptoms (*p* = 0.115), and phobic anxiety symptoms (*p* = 0.066; Figure 2 provides a graphical representation of these differences between mean scores).

## Discussion

The SCL-90-R (Derogatis, 2017) is one of the most internationally used questionnaire to assess psychological distress, especially in clinical practice (Parker et al., 1990; Weathers et al., 1996; Prunas et al., 2012; Gül et al., 2015; Christensen et al., 2016). However, it is a time-consuming questionnaire, and this usually is a serious problem in non-psychiatric hospital settings, in which there is not much time available for the patient’s psychological assessment. Therefore, short versions were developed to overcome this problem (Müller et al., 2010). One of these is the SCL-K-9 version (Klaghofer and Brähler, 2001). The major purpose of the current research was to measure, for the first time, the general psychopathology in a sample of patients with different type of skin diseases using the Italian adaptation of SCL-K-9 (Imperatori et al., 2020), with a focus on gender differences. From our results, among patients with skin diseases, women had higher levels of psychological distress than men, as previously reported in the literature, with data from different assessment tools (Finzi et al., 2007; Mina et al., 2015; Masood et al., 2016). This greater vulnerability experienced by women compared to men seems to have multifactorial and unclear reasons (Zachariae et al., 2004). For example, as asserted by Picardi et al. (2001b), in women, but not in men, the prevalence of psychiatric morbidity was higher in patients with lesions on visible parts of the body. According to the authors, the increased psychological vulnerability of female patients might be related to a higher impact of changes in body image on self-esteem compared to men (Picardi et al., 2001b). Particularly, the skin plays an essential role throughout the life cycle not only as a sensory organ, but even in socialization processes. So, skin appearance can affect body image and self-esteem through processes of stigmatization and lasting emotions like shame (Lahousen et al., 2016; Hawro et al., 2017). This interpretation is supported by other studies that stressed the concept that women are more invested in appearance (Pingitore et al., 1997) and less satisfied with their body image compared to men (Smith et al., 1999), but a more extensive investigation is needed to better understand sociological, anthropological, and psychological reasons beyond these observations.

Because of these previously reported significant differences in psychopatologic symptoms among women and men, confirmed by our own findings in the present study, it seems appropriate to us to have identified two different GSI cut-offs, which may be of help in detecting gender-specific psychopathology risk among dermatologic patients. Previous studies tested the SCL-K-9 in different clinical settings. For example, Goetzmann et al. (2009) investigated the psychological processing of transplantation in lung recipients and, among others psychometric tools, they used the German version of SCL-K-9; and Crössmann et al. (2010) used SCL-K-9 to evaluate an intervention to reduce psychopathology and improve quality of life in patients with an implantable cardioverter defibrillator. However, to the best of our knowledge, no research has focused on the investigation of the SCL-K-9 performance among dermatological patients, and on evaluating gender differences when measuring psychopathology with the SCL-K-9.

As for the ROC curve methodology, Imperatori et al., used the Italian version of the SCL-K-9 to discriminate between overweight/obese patients with and without significant binge eating disorder. The authors identified a cut-off of 0.83 or higher to categorize individuals as having binge eating disorder (Imperatori et al., 2020). Bianciardi et al. (2021) used the Italian SCL-K-9 to assess the level of psychopathology in women eligible for bariatric surgery, and identified a different cut-off (i.e., 0.50 or higher to identify patients with at least one psychiatric disorder from those without any psychiatric disorder) (Bianciardi et al., 2021). However, all participants of both studies were females.

One of the most used questionnaires to assess minor psychiatric disorders and to screen for depression symptomatology in dermatology is the GHQ-12 (Picardi et al., 2001a), for which a cut-off score of >7 has been used to identify symptoms of depressive disorders (Picardi et al., 2004). However, this questionnaire does not permit the ability to investigate other psychopathological issues that are detectable in dermatological patients and that can significantly impair their quality of life (Graham, 1955; Gupta and Gupta, 2013; Dalgard et al., 2015; Peters, 2016). For this reason, a SCL-K-9 single-item analysis has been performed, to highlight specific contents related to the psychological distress reported by patients (Klaghofer and Brähler, 2001; Imperatori et al., 2020), differentiating them according to gender.

As conceptualized by Derogatis and Cleary (1977), the Hostility dimension of SCL-90 (item #24) and first item of the SCL-K-9, reflects tendencies of externalized anger and aggressiveness; the Obsessive–compulsive dimension of SCL-90 (item #28), second SCL-K-9 item, reflects the tendency to worry and control the external and internal environments; the Depression dimension of SCL-90 (#31), third item of SCL-K-9, refers to depressive symptomatology (low or discouraged mood resulting from disappointment or imaginary or real loss); the Interpersonal sensitivity dimension of SCL-90 (#34), fourth SCL-K-9 item, is consistent with the traditional notion of the “inferiority complex,” and highlights feelings of personal inadequacy, self-deprecation, and acute self-consciousness; the Paranoia dimension of SCL-90 (#43), fifth SCL-K-9 item, closely reflects tendencies of suspiciousness; the Anxiety dimension of SCL-90 (#57), sixth SCL-K-9 item, leads to physical sensation and cognitive tendencies of being frightened and fearful; the Somatization dimension of SCL-90 (#58), seventh item of SCL-K-9, has a primary focus on psychological distress that arises from perceptions of bodily dysfunction; the Phobic Anxiety dimension of SCL-90 (#75), eighth SCL-K-9 item, has been designed to reflect the “agoraphobic” symptomatology; the Psychoticism dimension (#77), the last SCL-K-9 item, attempts to capture the symptoms of interpersonal distance and alienation.

As already noted, women reported significantly higher scores indicative of psychological distress than men, especially in some symptomatic areas. For this reason, and particularly in women who reach the SCL-K-9 cut-off, a more in-dept psychological assessment should be carried out, to evaluate levels of hostility, interpersonal sensitivity, obsessive–compulsive traits, depression, anxiety, and somatization symptoms, as well as psychoticism that could be detected but not fully identified as assessed through a brief-screening tool. These issues experienced by patients could affect general well-being and functioning, often causing suffering, isolation, and a worse adherence to treatments (Renzi et al., 2002; Picardi et al., 2006b; Yélamos et al., 2015) It is important to note some issues limiting the generalizability of our findings. First, the ratio among women and men in the sample was quite unbalanced; however, it reflects the greater access of women to Dermatology Units than men. To this respect, it is interesting to note that the Italian version of the SCL-K-9 had never been used, before the present study, to detect psychopathologic symptoms in men. Second, even though we excluded people reporting the presence of a major neuropsychiatric diagnosis, no *a priori* psychological evaluation has been carried out on our sample.

In conclusion, even if the SCL-K-9 is a screening and not a diagnostic tool, with its compact format could provide, in a short time, a wide range of information related to critical areas that challenge the mental health of patients with skin diseases. Moreover, it can allow to increase attention to this key area of concern that is often overlooked in dermatologic patients.

## Data availability statement

The raw data supporting the conclusions of this article will be made available by the authors, without undue reservation.

## Ethics statement

The studies involving human participants were reviewed and approved by Institutional Ethical Committee of dermatological research hospital IDI-IRCCS, Rome, Italy. The patients/participants provided their written informed consent to participate in this study.

## Author contributions

TS, DA, GC, and VA: Conceptualization. TS, GC, and PS: Enrollment and data curation. TS and DA: Methodology and formal analysis. DA, AG, and VA: Project administration and supervision. TS: Writing - original draft. TS, DA, GC, VA, and AG: Writing - review and editing. All authors discussed the results and contributed to the final manuscript.

## Funding

This study was supported in part by “Progetto Ricerca Corrente 2020–2021” of the Italian Ministry of Health.

## Conflict of interest

The authors declare that the research was conducted in the absence of any commercial or financial relationships that could be construed as a potential conflict of interest.

## Publisher’s note

All claims expressed in this article are solely those of the authors and do not necessarily represent those of their affiliated organizations, or those of the publisher, the editors and the reviewers. Any product that may be evaluated in this article, or claim that may be made by its manufacturer, is not guaranteed or endorsed by the publisher.
